# Effects of microscopic testa color and morphologyon the water uptake ability and drought tolerance of germination-stage rapeseed (*Brassica napus* L.)

**DOI:** 10.1080/21655979.2021.2000789

**Published:** 2021-12-24

**Authors:** Zong He Zhu, Abdul Sami, Zhi Peng Chen, Maliha Fatima, Wen Yin Zheng, Qing Qing Xu, Yu Hang Lei, Xue Zhi Jin, Hong Zhang, Yong Li, Yan Yu, Ke Jin Zhou

**Affiliations:** a College of Agronomy, Anhui Agricultural University, Hefei, China; b Department of Agronomy, University of Agriculture Faisalabad, Faisalabad, Pakistan

**Keywords:** Drought, imbibition, papillae, seed coat, seed color, testa

## Abstract

Drought is one of the most important abiotic stressors that affect crop yield. Therefore, the aim of the present study was to investigate correlations between germination-stage drought tolerance and the microscopic testa (i.e., seed coat) characteristics (color and papilla morphology) and imbibition abilities of 35 rapeseed (*Brassica napus* L.) accessions. After 2 h imbibition, seed water uptake (fresh weight increase) was significantly positively correlated with testa hue (*H*_HSB_), brightness (*B*_HSB_,), blue (*B*_RGB_), and lightness (L*), with correlation coefficients of 0.38, 0.34, 0.53, and 0.36, respectively, and significantly negatively correlated with saturation (*S*_HSB_), greenness-redness (a*), blueness-yellowness (b*), magenta (*M*), and yellow components (*Y*), with correlation coefficients of −0.53, −0.40, −0.53, −0.39, and −0.55, respectively. Furthermore, 5-h seed water uptake was significantly positively correlated with number of papillae (No.P), mean papillae area (APA), the papillae area ratio (PAR), gray value of red channel of papillae, with correlation coefficients of 33, 0.36, 0.43, and 0.43, respectively. Under drought conditions, genotypes with more rapid water absorption exhibited higher germination rates and stronger drought tolerance, and the germination rate and drought tolerance of black-seeded accessions were highest, followed by red-seeded accessions and then yellow-seeded accessions, which exhibited the lowest germination rate and drought tolerance. Germination rate was significantly negatively correlated with *B*_RGB_, *H*_HSB_, L*, D_g_, and *D*_b_ and significantly positively correlated with *S*_HSB_ and *Y*, regardless of drought conditions. At the germination stage, *D*_b_TP was negatively correlated with drought tolerance.

## Introduction

The genus *Brassica* L. (Cruciferae) contains several economically important species, including *Brassica campestris* L. (low yield and poor resistance to stress), *Brassica juncea* L. (low yield and strong resistance), and *Brassica napus* L. (high yield and strong resistance). Rapeseed (i.e., *B. napus*) is one of the world’s most important oilseed crops [1,2]. China is one of the world’s largest rapeseed producers, with an annual cultivation area of ~7,000,000 ha and production of ~13,000,000 tons (in 2005), which accounts for about a third of total global production [3,4–9]. However, the total cultivation area allocated to rapeseed has been decreasing in China since 2016 and drought is one of the main reasons. In China, more than 85% of rapeseed production occurs in the Yangtze River Basin [10].

Drought is one of many environmental factors that seriously affect plant growth and development. Drought can have serious consequences in both developed and developing countries, and their frequency, severity, and duration are increasing [11]. The growing biophysical vulnerability contexts and intensity in Asian Least developed countries (LDCs) negatively impact food security, human health, biodiversity, water resources, hydroelectric power generation, streams, perennial springs, and livelihoods. In the monsoon climatic zone, South and Southeast Asian LDCs, such as Bhutan, Bangladesh, Nepal, Cambodia, and Lao PDR, have also experienced increasing droughts, which are caused by changes in the timing and distribution patterns of precipitation. South Asia has been recognized as among the world’s most drought-prone regions. Over the last five decades, drought has been reported at least once every three years in India, Pakistan, Afghanistan, Sri Lanka, Bangladesh, and Nepal [12,13].

Temperature, inadequate humidity, biological pollution, solar radiation, wind, chemical pollution, wildfires, and natural disturbances also affect the sensitivity of plants to stressors [12,14].

The negative effects of drought events are typically addressed by improving industrial structure and ecological environment, improving farming systems, changing crop composition, breeding drought-tolerant crop varieties, fully utilizing effective precipitation, improving irrigation facilities, and applying water-saving technologies [11,14]. In this area, seasonal precipitation is unevenly distributed, and 60–80% of the annual rainfall occurs during summer. Therefore, rapeseed, which is typically sown during autumn, often encounters drought stress, which ultimately results in resulting in reduced seedling emergence and vigor, and improving both the germination rate of rapeseed during drought conditions and germination-stage drought tolerance are important strategies for countering drought event during autumn. Seed germination is the most sensitive developmental stage in regard to drought stress [15,16], and drought is one of the main environmental factors that affect seed germination and plant growth [16,17].

Nitric oxide, cyanide, and karrikin1 have been reported to induce seed germination and stimulate the production of ethylene, 1-aminocyclopropane-1-carboxylic acid (ACC), ACC oxidase (ACO), and ACC synthase (ACS) [1,2,16]. Melatonin has also been reported to increase seedling growth, catalase, peroxidase, ascorbate peroxidase, proline, chlorophyll, and anthocyanin content, as well as photosynthesis rate, under aluminum and cadmium stress [16]. Under low temperature and drought conditions, appropriate concentration seed priming such as salicylic acid (SA), gibberellic acid (GA), sodium nitroprusside, calcium chloride(CaCl_2_) and abscisic acid (ABA) significantly improved germination potential, germination rate, germination index, fresh stem weight, dry stem weight, stem length, and seed vigor index [14].

As a result of frequent seasonal (autumn) drought and inadequate irrigation facilities, rapeseed production in the Yangtze River basin often suffers from low germination rates and poor seedling quality and, consequently, suffers from relatively low seed yield and reductions in seeded area [12].

Seed germination is a fundamental process in plant growth and development. The germination process is initiated by the uptake of water (i.e., imbibition), followed by increases in respiratory and enzymatic activities, consequent reactivation of metabolism, and, ultimately, emergence of the radicle [18]. Imbibition is a physical process that mainly involves the rapid uptake of water [19,20] and plays a critical role in seed germination since it is essential for the activation of enzymes, decomposition of starch into sugar, and transport of nutrients to the developing embryo [21,22].

$$Starch\;\left[{\left(C_{6}H_{10}O_{5}\right)}_{n}\right]+nH_{2}{O^{\beta }}^{-amylase}maltose{\left(C_{12}H_{22}O_{11}\right)}^{\alpha -\;glucosidase}glucose\;\left(nC_{6}H_{12}O_{6}\right)$$

Seeds are particularly stress-sensitive during seed imbibition [23]. Because water restriction affects water uptake and seed germination ability, adequate water availability is a critical factor during the germination stage [17]. These traits affect the effective absorption of water, directly or indirectly, on the germination and drought tolerance of rapeseed seeds. Water uptake rate is important for seed germination and is strongly affected by water permeability, which is influenced by seed shape, structure, composition, and initial water content [24]. Genetic factors play a significant role in imbibition and germination [24,25], as do seed coat features.

The testa (i.e., seed coat) is the primary barrier to water penetration during imbibition, and both seed imbibition and germination are significantly affected by testa thickness, density, cell wall thickening, and chemical composition (e.g., presence of tannins, pigments, crystal substances) [10,26,27]. For example, dark-colored seeds have been reported to exhibit slower water absorption [10,26–28], and yellow-seeded rapeseed accessions exhibit more rapid water uptake than red- or black-seeded accessions [10], whereas black-seeded accessions exhibit higher germination rates, emergence percentages, and seedling establishment than dark and light brown-seeded accessions [10]. Testa color determines seed composition, which, in turn, affects seed germination and seedling growth [29].

Studies that have investigated relationships between testa color, water absorption rate, and seed germination have mainly focused on testa color classification [10], and relationships between rapeseed testa microstructure and color, seed imbibition, and germination-stage drought tolerance have yet to be reported. The aims of the present study were (a) to analyze the imbibition ability and germination-stage drought tolerance of rapeseed accessions and (b) to assess the associations of these traits with testa microstructure and color.

## Methods and materials

### Materials

Seeds of 35 rapeseed (*Brassica napus* L.) germplasm accessions (Table 1) were selected and collected from winter rapeseed cultivation areas in China, including Huang Huai and areas surrounding the upper, middle, and lower Yangtze River. After air-drying, the seeds were stored with silica gel.

**Table 1.** Sources and characteristics of rapeseed (*Brassica napus* L.) germplasm accessions used in the present study

Code	Line	Origin	Name	Type	Seed color	Papilla type
1	Bn07005	YHR	Huang C	genetic material	red	LBP
3	Bn07009	YHR	Shanyou No.10	bred variety	red	MBP
4	Bn07010	YHR	Shanyou No.9	bred variety	black	LSP
5	Bn07011	MYR	Huayouza No.7	bred variety	red	LSP
6	Bn07013	MYR	Huahuang No.1	bred variety	yellow	LBP
7	Bn07014	LYR	Huyou No.15	bred variety	red	LBP
10	Bn07019	UYR	Yuhuang No.1	bred variety	yellow	MSP
12	Bn07021	LYR	Huawanyou No.2	bred variety	red	MBP
13	Bn07022	MYR	Huayou 2008	bred variety	black	LBP
14	Bn07023	MYR	Zhongshuang No.11	bred variety	black	LBP
16	Bn08010	MYR	Zhongshuang No.9	bred variety	black	LBP
17	Bn08011	LYR	Huyou No.18	bred variety	black	MBP
18	Bn08012	LYR	Ningza No.11	bred variety	red	LBP
19	Bn08013	MYR	Fengyou701	bred variety	black	LSP
20	Bn08014	MYR	Xiangzayou No.6	bred variety	black	LSP
21	Bn08015	LYR	Wanyou No.17	bred variety	black	MBP
22	Bn08016	MYR	H9954	genetic material	black	MSP
24	Bn08035	LYR	Zheyou 50	bred variety	black	MSP
25	Bn08036	LYR	Huaiza No.3	bred variety	black	MSP
27	Bn08037	YHR	Yuyou No.5	bred variety	black	LBP
28	Bn08038	YHR	Qinyou No.9	bred variety	red	LBP
32	Bn08043	UYR	Youyan No.10	bred variety	yellow	MSP
33	Bn08044	MYR	Zhongyou012	bred variety	black	LSP
34	Bn08045	UYR	G2153	genetic material	black	LSP
36	Bn08047	MYR	Zhongyouza No.2	bred variety	black	LSP
37	Bn08048	YHR	Qinyan 211	bred variety	black	MSP
39	Bn08110	MYR	Zhongyouza No.8	bred variety	red	MSP
40	Bn08112	MYR	Xiwang 98	bred variety	red	LSP
42	Bn08113	MYR	Zhongyouza No.7	bred variety	black	LBP
43	Bn08114	MYR	Huayouza No.10	bred variety	red	MBP
44	Bn08115	YHR	Qinyou No.6	bred variety	red	LSP
45	Bn08116	MYR	Huayouza No.9	bred variety	black	MSP
47	Bn08119	MYR	Zhongyouza No.11	bred variety	black	MSP
48	Bn08121	MYR	H4270	genetic material	black	MSP
49	Bn08132	LYR	Hongyou No.3	bred variety	black	MSP

UYR: upper Yangzi River; MYR: middle Yangzi River; LYR: lower Yangzi River; YHR: Yellow and Huai River; MBP: more and bigger papilla; LBP: less and bigger papilla; MSP: more and smaller papilla; LSP: less and smaller papilla.

### Microstructure and color analysis

**Figure 1.**
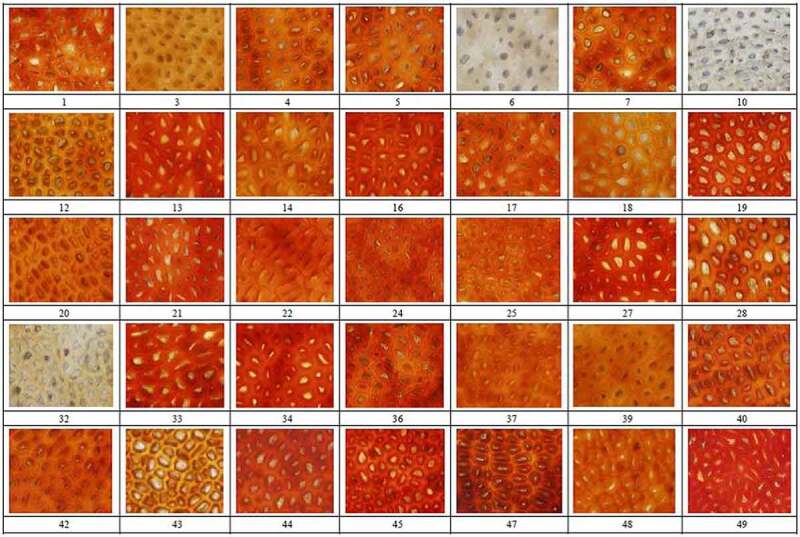
Microscopic color and morphology of testa from 35 rapeseed (*Brassica napus* L.) accessions from China

The testa was manually cut, and photomicrographs were collected using an DP70 digital camera (OLYMPUS, JAPAN) (aperture set to a maximum of 6 and magnification of 400 times; Figure 1).

The microscopic color of the testa images was assessed using four color spaces: *RGB* (red, green, and blue) in which *R*_RGB_, *G*_RGB_, and *B*_RGB_ were in the range 0–255, *RGB* gray values (gray values of the red, green, and blue channels [*D*_r_, *D*_g_, and *D*_b_, respectively]). Hue saturation and brightness (HSB), in which *H*_HSB_ was defined as the spectral values of different wavelengths, ranging from 0 to 360, where 0 and 360 were red, and every 60 were successively yellow, green, cyan, blue, and magenta, *S*_HSB_ was defined as color depth, ranging from 0% to 100%, *B*_HSB_ was defined as color brightness, ranging from 0% to 100%, CIE L*a*b*(lightness greenness-redness and blueness-yellowness), in which L* values ranged from 0 (black) to 100% (white), *a* represents the range from red to green, *b* represents the range from blue to yellow, the value range of *a* and *b* were −120 to +120), and CMYK (*C*, cyan; *M*, magenta; *Y*, yellow; *K*, black). The HSB, L*a*b*, and CMYK values were measured using image analysis software (Photoshop CS v.6.0, Adobe USA) and the software (Image Pro-Plus v.6.0 Media Cybernetics, USA), was used to measure *RGB* gray values . The color values of each accession were calculated as the mean values of five individual images of five single seeds, which were repeated three times using appropriate image analysis software (Image Pro- Plus v.4.5.

For microstructure analysis, papillae area was calculated using the density tools in Quantity one (×400 calculates microscopic papillae area). The papillae area ratio (PAR) was defined as the proportional area of each micrograph represented by papillae, whereas average papillae area (APA) was calculated as the average of 50 papillae, and the number of papillae (No.P) in each micrograph was counted manually. Mean values for each of the seed characteristics were calculated using five individual seeds, with three replicates each. *IOD*, integrated optical density; *D*_r_P, red channel gray value of papilla; *D*_g_P green channel gray value of papilla; *D*_b_P, blue channel gray value of papilla; *D*_g_, green channel gray value of; *D*_b_, blue channel gray value of testa. Average papillae area.

### Water uptake analysis

For each accession, 500 seeds (with three replicates per accession) were weighed and then placed in 100-ml plastic centrifuge tubes with 50 ml room temperature (25°C) distilled water. The seeds were removed from the water after 1, 2, 3, 4, 5, and 24 h, blotted dry, and weighed. Water uptake was then calculated as mean percent weight increase (n = 3) [30].

### Conductivity analysis

For each accession, 1 g seeds were placed in plastic centrifuge tubes with 100 ml room temperature (25°C) deionized water. The seeds were removed after 1, 2, 3, 4, 5, and 24 h, and then electrical conductivity of seed imbibition was measured using a conductivity meter (DDS-12D, Hongyi Shanghai, China) as described previously [31].

### Evaluation of drought tolerance

For each accession, 100 randomly selected seeds were weighed, soaked in 1% sodium hypochlorite solution for 5 min, and rinsed three times using distilled water. The sterilized seeds were then dried at room temperature (25°C) until reaching their initial weight. Drought conditions were simulated using 10% PEG-6000, as described previously [32], and controls were also included. Seed germination was evaluated by incubating seeds between wet filter paper in Petri dishes (25 × 25 cm) for 7 d and then employing the International Seed Testing Association (ISTA) vitality test method [33]. Germination rate, seedling height, and fresh seedling weight were recorded, and relative germination rate, relative seedling height, and relative seedling fresh weight were calculated as percentages of the control seeds and seedlings. Finally, drought tolerance (drought tolerance index, DTI) was calculated as the product of relative germination rate and relative seedling height [34].

### Statistical analysis

The experiment was conducted using a completely randomized design, and all experiments were performed using at least three replicates per accession. Microsoft Office Excel (Microsoft Co., 2007) was used for primary data analysis. Percentage data were subjected to arcsine transformation, and the significance of differences between means was calculated using Duncan’s new multiple range test (*P* < 0.05). Analysis of variance (ANOVA) and correlation analyses were conducted using SPSS10. Principle component analysis (PCA) was performed using the FactoMineR package (version 2.1). Principal component analysis can extract less and uncorrelated comprehensive indices from measured variables and circumvent problems caused by collinearity among variables. The Pearson correlation analysis method was used to screen indices for significant correlations with germination rate, seedling height, fresh seedling weight, relative germination rate, relative seedling height, and DTI. Principal component analysis was applied to 12 of the 26 testa color and papilla trait indices.

## Results

### Seed characteristics

The 35 rapeseed accessions were categorized as black (21 accessions), red (11 accessions), or yellow (3 accessions) by eye (Table 1), and based on the number and size of papillae, were also categorized as MBP (more and bigger papilla, five accessions), LBP (less and bigger papilla, 10 accessions), MSP (more and smaller papilla, 11 accessions), or LSP (fewer and smaller papillae, 9 accessions; Table 1, Figure 1).

The microscopic color parameters and papillary indices varied significantly between varieties, and the calculated coefficient of variation values ranged from 1.05% to 152.85% (Table 2). For color, the greatest and smallest coefficient of variation values were observed for *D*_b_ (152.85%) and *D*_r_ (1.05%), respectively, and for papilla traits, the greatest and smallest coefficient of variation values were observed for PAR (37.45%) and P (24.40%). In general, the coefficients of variation values were relatively large, which indicated significant differences in the color and papilla traits of the rapeseed accessions.

**Table 2.** Micromorphological testa traits of 35 rapeseed (*Brassica napus* L.) germplasm accessions from China

Index	Min	Max	Mean	STD	*CV* (%)	Index	Min	Max	Mean	STD	*CV* (%)
*H*_HSB_	75.51	171.22	93.71	21.97	23.44	*D*_r_	182.66	192.09	188.50	1.98	1.05
*S*_HSB_	77.87	174.99	101.19	22.99	22.72	*D*_g_	38.19	173.58	77.56	29.43	37.94
*B*_HSB_	0.21	0.99	0.89	0.17	19.19	*D*_b_	2.35	148.66	18.43	28.17	152.85
*R*_RGB_	149.60	205.20	190.39	10.21	5.36	IOD	110,462,000.00	237,129,000.00	132,056,857.1	25,570,052.00	19.36
*G*_RGB_	33.40	328.40	82.24	52.73	64.12	No.P	193.00	710.00	490.31	119.65	24.40
*B*_RGB_	1.40	151.20	21.51	31.47	146.30	APA	6.60	30.90	15.49	5.28	34.09
L*	33.60	72.60	49.19	7.47	15.19	PAR	10.30	38.50	19.04	7.13	37.45
a*	2.60	56.60	43.48	13.15	30.24	*D*_r_P	149.95	210.79	195.46	9.55	4.89
b*	14.60	59.00	52.44	9.34	17.81	*D*_r_TP	36230.64	141,568.10	95,737.96	23,271.76	24.31
*C*	0.25	0.45	0.31	0.04	12.90	*D*_g_P	32.30	175.33	102.17	35.53	34.78
*M*	0.31	0.97	0.80	0.15	18.75	*D*_g_TP	9721.52	83,089.91	49,522.07	18,711.41	37.78
*Y*	0.41	1.00	0.94	0.13	13.83	*D*_b_P	1.05	150.29	35.17	34.25	97.38
*K*	0.00	0.16	0.02	0.03	140.00	*D*_b_TP	202.06	51,784.09	16,235.34	13,095.38	80.66

*P < 0.05, ** *p < *0.01) *H*_HSB_, hue of testa; *B*_HSB,_ brightness of testa; *S*_HSB_, S of testa; *B*_RGB_, B value of RGB; *G*_RGB_, G value of RGB; L*, lightness; a*, from magenta to green; b*, from yellow to blue; *M*, magenta; *Y*, yellow; *D*_b_, blue channel gray value of testa; *IOD*, integrated optical density; *D*_r_P, red channel gray value of papilla; *D*_g_P, green channel gray value of papilla; *D*_b_P, blue channel gray value of papilla; *D*_g_, green channel gray value of; *D*_b_, blue channel gray value of testa; No.P, number of papillae; APA, average papillae area; PAR, papillae area ratio.

### Water uptake

**Figure 2.**
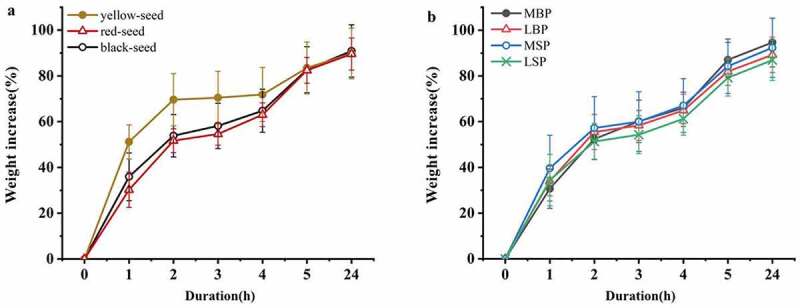
Effect of testa color and morphology on the water uptake rate of rapeseed accessions from China. (a) Effect of testa color. (b) Effect of testa morphology

Most accessions exhibited rapid water uptake, with fresh weights increasing by >80% within 5 h (Table 3). Over the full 24 h, the imbibition process could be separated into four distinct stages: fastest period (phase I, 0–2 h), steady period (phase II, 2–4 h), sub-fast period (phase III, 4–5 h), and slowest period (phase IV, 5–24 h). In phases I and II, the imbibition rates of the yellow-seeded accessions were significantly greater than those of either the red- or black-seeded accessions, and in phases III and IV, the imbibition rates of the yellow- and black-seeded accessions were greater, although not significantly (Figure 2(a)). Furthermore, the imbibition rates of the MSP and MBP accessions were greatest during phases I and II and phases III and IV, respectively, whereas the rates of the LSP accessions were lowest during all four phases and were significantly lower than those of the MSP and MBP accessions during phases I and II and phases III and IV, respectively (Figure 2(b)).

**Table 3.** Effect of imbibition duration on the water uptake and electrical conductivity of seed from rapeseed (*Brassica napus* L.) germplasm accessions from China

	Increase in fresh weight (%)								Electric conductivity (μSg^−1^cm^−1^)					
Accession	1 h	2 h		3 h	4h	5 h		24 h	1 h	2 h	3 h	4 h	5 h	24 h
1	21.0	49.2		54.2	67.4	84.3		89.5	37.2	98.6	121.0	140.1	170.4	243
3	16.8	52.7		54.7	58.6	79.1		79.2	29.7	56.2	61.7	86.6	110.0	160
4	21.5	47.2		51.6	55.8	73.2		79.5	18.8	37.4	46.1	58.0	75.3	119
5	18.8	46.0		47.3	57.0	73.6		81.9	14.7	32.3	41.2	51.7	65.7	111
6	46.0	75.7		76.1	77.5	78.1		85.2	136.8	158.3	176.6	196.2	271.0	322
7	31.8	56.3		57.5	61.7	79.0		83.6	48.6	99.7	101.6	133.0	155.1	235
10	47.8	56.5		57.2	58.3	75.8		81.8	115.7	126.2	139.1	145.4	150.6	162
12	28.8	53.7		58.7	64.3	84.2		96.6	14.2	36.5	45.6	60.9	79.8	132
13	30.9	51.2		55.8	68.2	88.5		99.8	41.7	77.4	81.5	85.1	99.3	146
14	29.9	57.0		59.4	64.8	80.5		86.4	24.4	40.0	52.7	68.5	94.7	151
16	34.6	57.8		59.4	64.7	86.3		97.5	25.6	46.1	47.5	59.7	74.5	128
17	38.9	53.9		76.5	77.0	99.7		109.2	44.8	67.5	80.9	101.3	124.4	221
18	37.5	53.9		56.7	73.2	92.9		101.7	42.5	59.0	71.9	86.5	131.2	184
19	33.6	50.1		58.3	61.0	81.9		88.5	43.2	52	63.2	75.9	108.7	161
20	45.8	62.7		66.9	74.3	94.1		104.9	54.6	89.1	95.3	110.2	137.9	198
21	35.2	52.1		56.1	67.3	84.9		93.5	25.1	38.3	53.0	70.4	109.5	162
22	40.2	59.5		63.7	77.0	97.1		107.7	38.4	52.8	70.8	98.9	122.5	182
24	15.5	53.8		58.0	72.2	89.4		105.9	24.5	35.5	45.4	58.0	71.3	120
25	13.8	27.2		32.3	41.4	57.7		65.7	29.2	40.8	54.7	70.5	84.1	130
27	35.2	51.8		55.6	56.2	73.5		80.3	27.9	32.9	47.5	64.1	80.1	120
28	37.6	49.9		54.3	54.6	74.8		81.2	63.5	78.1	83.4	99.3	108.6	154
32	59.7	76.7		78.1	79.8	96.5		102.4	152.1	186.3	193.6	201.7	228.2	237
33	54.7	65.6		67.7	69.6	84.9		90.3	54.5	65.2	88.2	100.6	130.5	197
34	36.6	50.3		50.6	50.8	67.6		74.6	67.7	71.5	83.5	85.2	115.7	172
36	30.6	45.8		47.7	58.5	74.6		81.6	32.1	49.8	54.1	65.1	77.3	114
37	30.6	39.4		42.8	58.0	74.0		79.6	18.1	36.3	37.5	44.7	54.3	88
39	37.8	62.8		64.1	69.0	88.0		95.7	56.8	76.1	108.2	133.7	157.6	233
40	29.6	41.8		45.0	60.2	78.9		89.5	42.8	43.5	54.6	67.9	81.1	135
42	36.3	51.9		54.6	61.3	81.3		87.1	61.4	105.5	80.0	95.6	112.7	160
43	33.9	49.9		54.7	62.9	87.3		94.6	104.5	108.2	108.7	109.0	124.9	164
44	38.8	52.1		53.8	64.4	84.3		91.6	62.7	73.4	93.5	109.5	125.9	170
45	48.6	63.2		68.7	70.7	86.1		92.5	108.2	109	110.4	123.0	162.5	209
47	48.6	58.2		59.7	65.9	85.5		95.7	81.7	98.5	109.0	124.8	144.5	180
48	45.2	61.7		61.9	62.5	80.1		86.1	55.9	67.4	79.2	100.1	109.7	162
49	48.3	70.7		74.1	82.6	96.5		102.4	57.7	72.3	80.9	98.2	107.1	153
*mean*	35.4	54.5		58.1	64.8	82.7		90.4	53.1	71.9	81.8	96.6	118.8	169
*F* value														
*H*_HSB_	0.25	0.38*		0.28	0.16		−0.04	−0.1	0.34*	0.35*	0.44**	0.45**	0.46**	0.43**
*S*_HSB_	−0.42*	−0.53**	−0.48**		−0.38*		−0.14	−0.07	−0.62**	−0.59**	−0.70**	−0.66**	−0.67**	−0.63**
*B*_HSB_	0.18	0.34*	0.24		0.13		−0.04	−0.1	0.28	0.32	0.40*	0.43**	0.43**	0.41*
*G*_RGB,_	0.09	0.18	0.16		0.13		0.12	0.05	0.48**	0.44**	0.46**	0.40*	0.38*	0.34*
*B*_RGB_	0.40*	0.53**	0.48**		0.38*		0.13	0.06	0.63**	0.60**	0.70**	0.67**	0.68**	0.64**
L*	0.17	0.36*	0.36*		0.27		0.09	0	0.39*	0.43**	0.54**	0.54**	0.57**	0.60**
a*	−0.25	−0.40*	−0.34*		−0.24		−0.09	0.01	−0.47**	−0.49**	−0.60**	−0.59**	−0.59**	−0.58**
b*	−0.48**	−0.53**	−0.45**		−0.34*		−0.1	−0.05	−0.65**	−0.59**	−0.67**	−0.62**	−0.62**	−0.53**
*M*	−0.19	−0.39*	−0.36*		−0.26		−0.1	−0.02	−0.40*	−0.46**	−0.56**	−0.55**	−0.56**	−0.56**
*Y*	−0.42*	−0.55**	−0.50**		−0.41*		−0.16	−0.11	−0.58**	−0.56**	−0.68**	−0.65**	−0.68**	−0.64**
*D_b_*	0.24	0.25	0.17		0.02		−0.19	−0.21	0.36*	0.33*	0.37*	0.37**	0.40*	0.35*
*IOD*	0.16	0.2	0.15		0.01		−0.19	−0.21	0.29	0.31	0.33*	0.34*	0.38*	0.34*
No.P	0.21	0.16	0.22		0.22		0.33*	0.35*	0.11	013	0.1	0.08	0.09	0.07
*D*_r_P	0.18	0.16	0.24		0.34*		0.43**	0.39*	0.06	0.22	0.23	0.23	0.25	0.3
APA	−0.01	0.12	0.21		0.29		0.36*	0.31	0.06	0.29	0.27	0.27	0.32	0.37*
PAR	−0.01	0.12	0.24		0.34*		0.43**	0.39*	0.06	0.07	0.22	0.23	0.25	0.3

*P < 0.05, ** *p < *0.01) *H*_HSB_, hue of testa; *B*_HSB,_ brightness of testa; *S*_HSB_, S of testa; *B*_RGB_, B value of RGB; *G*_RGB_, G value of RGB; L*, lightness; a*, from magenta to green; b*, from yellow to blue; *M*, magenta; *Y*, yellow; *D*_b_, blue channel gray value of testa; *IOD*, integrated optical density; *D*_r_P, red channel gray value of papilla; *D*_g_P, green channel gray value of papilla; *D*_b_P, blue channel gray value of papilla; *D*_g_, green channel gray value of; *D*_b_, blue channel gray value of testa; No.P, number of papillae; APA, average papillae area; PAR, papillae area ratio.

Correlation analysis indicated that 2-h water uptake (weight increase after 2 h imbibition) was significantly positively correlated with *H*_HSB_, *B*_HSB_, *B*_RGB_, and L*, with correlation coefficients of 0.38, 0.34, 0.53, and 0.36, respectively, and significantly negatively correlated with *S*_HSB_, a*, b*, M, and *Y*, with correlation coefficients of −0.53, −0.40, −0.53, −0.39, and −0.55. Meanwhile, 5-h water uptake was significantly positively correlated with the indices of papillae (No.P, *D*_r_P, APA, and PAR), and the correlation coefficients were 0.33, 0.43, 0.36,^,^ and 0.43, respectively.

### Electrical conductivity

**Figure 3.**
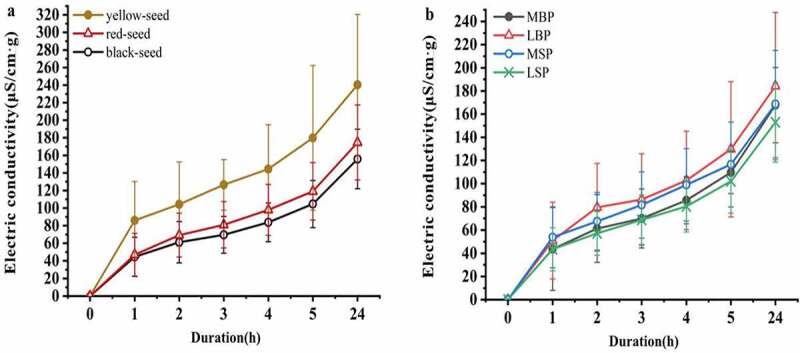
Effect of testa color and morphology on the rate of electric conductivity increase for rapeseed accessions from China. (a) Effect of testa color. (b) Effect of testa morphology

After 1 h imbibition, the three yellow-seeded accessions (Huahuang No.1, Yuhuang No.1, and Youyan No.10) exhibited the greatest electrical conductivity (>110 µSg^−1^cm^−1^), and except for Huayouza No.9 and Huayouza No.10, the red- and black-seeded accessions exhibited low electric conductivity (>70 µSg^−1^cm^−1^; Table 3). The mean electrical conductivity of the yellow-seeded accessions was significantly greater than that of either the red- or black-seeded accessions, regardless of soaking time. The mean electric conductivity of the black-seeded accessions was consistently lowest but not significantly lower than that of the red-seeded accessions (Figure 3(a)). After 2, 3, 4, 5, and 24 h imbibition, the mean electric conductivity of the LBP accessions was greatest, whereas that of the LSP accessions was lowest, and there were significant differences between the two groups (Figure 3(b)).

Correlation analysis showed that S_HSB_, a*, b*, M and Y were significantly negatively correlated with electrical conductivity after 3 h imbibition (P < 0.01), while H_HSB_, B_HSB_, D_b_, IOD, and the electric conductivity after 5 h imbibition was significantly (P < 0.05) or extremely significantly (P < 0.01) negatively correlated, and the correlation coefficient was the highest. With the exceptions of APA and electric conductivity after 24 h imbibition, electrical conductivity was significantly positively correlated with all the papilla indices (P < 0.05), and associations with the other indices were not significant.

### Drought tolerance

**Figure 4.**
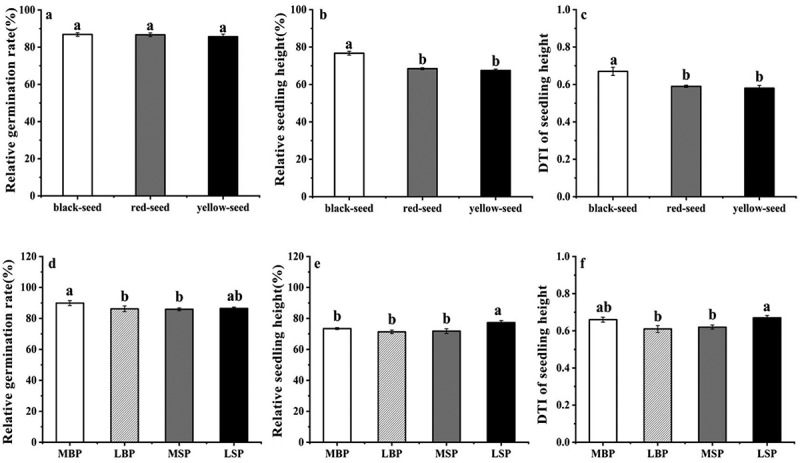
(a and d) Relative germination rate. (b and e) Relative seedling height. (c and f) DTI of seedling height

The mean drought tolerance index (DTI) was 0.63, and the accessions with the highest and lowest drought tolerance indices were Huyou No.18 (0.92) and Huyou No.15 (0.45), respectively (Supplementary Table S1). There was no significant difference in the relative germination rates of the black-, red-, and yellow-seeded accessions (Figure 4(a)). The relative seedling height and DTI values of the black-seeded accessions were considerably higher than those of the red- and yellow-seeded cultivars, and there was no significant difference between values for the red- and yellow-seeded accessions (Figure 4(b,c)). The relative germination rate values of the MBP accessions were significantly greater than those of the LBP and MSP accessions but not significantly different than those of the LSP accessions (Figure 4(d)). The relative seedling height values of the LSP accessions were greater than those of the MBP, LBP, and MSP accessions (Figure 4(d)), and the DTI values of the LSP accessions were significantly higher than those of the LBP and MSP accessions. However, there was no significant difference between the drought index tolerance of the LSP and MBP accessions or between the LBP and MSP accessions (Figure 4(f)).

Germination rates under normal and drought conditions were significantly (*P* < 0.05) or extremely significantly (*P* < 0.01) negatively correlated with the testa micro-color parameters *B*_RGB_, *H*_HSB_, L*, *D*_g_, and *D*_b_ and significantly (*P* < 0.05) or extremely significantly (*P* < 0.01) positively correlated with *S*_HSB_ and *Y*. The DTI and *D*_b_TP were significantly negatively correlated (*P* < 0.05).

Relative seedling height, relative fresh weight, and DTI were significantly positively correlated with 4-h water uptake (r = 0.61, *P* < 0.01). Electric conductivity after 1, 2, 3, 4, 5, and 24 h of imbibition was significantly correlated with the germination rate and relative germination rate under drought stress (*P* < 0.05) but not with DTI.

Germination rates under normal and drought conditions were negatively correlated with *B*_RGB_, *H*_HSB_, L*, *D*_g_, and *D*_b_ (*P* < 0.05) and significantly positively correlated with *S*_HSB_ and *Y* (*P* < 0.05). Seedling height under normal water availability conditions was significantly negatively correlated with testa *B*_RGB_ (*P* < 0.05) and significantly positively correlated with *S*_HSB_ and *Y* (*P* < 0.05), whereas seedling height under drought conditions was significantly negatively correlated with *H*_HSB_, *S*_HSB_, and L*(*P* < 0.05). In addition, fresh seedling weight under drought conditions was significantly negatively correlated with b*and *Y* (*P* < 0.05). Furthermore, *D*_g_P was significantly negatively correlated with germination rate under normal water availability (*P* < 0.05), and *D*_b_TP was significantly negatively correlated with seedling height, relative seedling height, and DTI under drought conditions (*P* < 0.05).

### Principal component analysis (PCA)

Principal component analysis of the 12 parameters of testas showed that the contribution rates of the first three principal components were 61.884%, 15.723%, and 8.820%, respectively, and the cumulative contribution rate was 86.428%, which basically included most of the information of the seed coat (supplementary Table S2). The feature vectors of *S*_HSB_, *H*_HSB_ and L* in the first principal component are relatively high, indicating that the three parameters of *S*_HSB_, *H*_HSB_ and L* in the first principal component account for the main factors; In the second principal component, the feature vectors of *D*_g_P and *D*_b_TP were higher, indicating that the two micro-color parameters were the main factors, and the feature vector of the blue channel gray value (*D*_b_) in the third principal component was higher, indicating that the blue channel gray value (*D*_b_) was the main factor in the third principal component.

**Figure 5.**
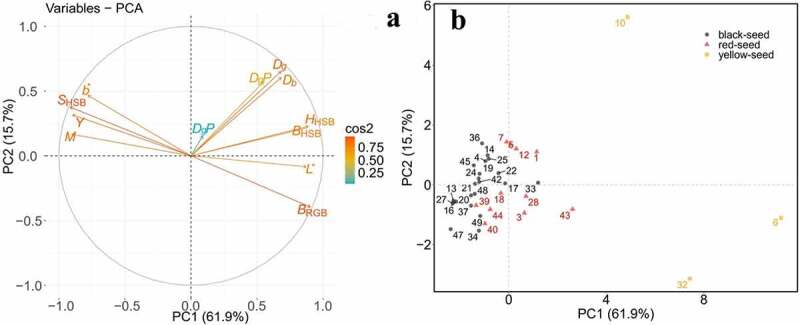
Principal component analysis of relationships among micromorphological testa characteristics. (a) Contribution of testa characteristics to two main principal components. (b) Grouping of accessions based on two main principal components

Positive correlations were found for the following indices: *H*_HSB_ and *B*_HSB_; L* and *B*_RGB_; *D*_g_P and *D*_g_, *D*_b_; *S*_HSB_ and b^*,^M,Y since the angle and directions between vectors is below 90° (Figure 5(a)). Negative correlations were observed between *H*_HSB_,*B*_HSB_, *B*_RGB_, and *S*_HSB_, b*,M,Y parameters. Negative correlations were also recorded between *S*_HSB_ and L*, D_g_, *D*_b_; L* and b*, M, Y parameters since the angle is higher than 90°and directions between vectors is below 90º. There was no correlation between *D*_b_P and all other parameters since the angle between the vectors was 90º.

Principal component analysis can distinguish rapeseed of yellow-seed, red-seed and black-seed. It was possible to select three groups of all lines (I and II) (Figure 5(b)). Group I with the higher PC1 comprised three yellow seed lines (6, 10 and 32), most of the red-seed rapeseed accessions (1,3,7,28,43, and 44) and four black-seed accessions (17,22,33 and 43). The accessions in group I had lower mean values of relative germination rate (with 85.62%), relative seedling height (with 67.02%) and DTI of seeding height (with 0.57) (supplementary Table S2), which could be recorded as more sensitive to drought stress. Group II with the lower PC1 consisted of other 22 accessions with the higher mean values of relative germination rate (with 87.34%), relative seedling height (with 77.02%) and DTI of seeding height (with 0.67) (supplementary Table S2) could be named as higher resistance to drought stress (Figure 5(b)).

## Discussion

### Seed characteristics

Testa color is one of the main contributing factors of imbibition rate in a variety of crop plants, including soybean, rapeseed, wheat, and chickpea [11,16,35,36]. Indeed, as in the present study, previous studies have reported that the imbibition rates of black-seeded accessions are higher than those of yellow-seeded accessions [26–28,37]. The present study’s finding that 2-h water uptake and electric conductivity were significantly positively correlated with both testa hue and brightness has also been reported previously [10].

In beans, black color has been reported to improve seed vigor and germination [30,38]. In the present study, the germination rates and seedling heights of black- and red-seeded accessions were significantly greater than those of yellow-seeded accessions, regardless of water availability, and black-seeded accessions exhibited significantly greater germination-stage drought tolerance than either the red- or yellow-seeded accessions. In the present study, the yellow-seed accession 6 exhibited relatively high drought tolerance during germination, which indicated that the germination-stage drought tolerance of yellow-seeded rape was relatively weak. However, yellow-seeded germplasm with strong germination-stage drought tolerance could still be selected and used to generate more drought-tolerant yellow-seeded varieties [39,40]. Drought resistance should be established to ensure yield stability under water scarcity. The resistance indices investigated in the present study is considered the most appropriate criteria for the assortment of high-yielding genotypes under drought stress [41].

### Seed imbibition and papillae

During imbibition, water is distributed into the crevices, cracks, and flaws of the testa and is then absorbed by seed tissues. Previous studies have demonstrated that the rate of water uptake during this phase is (1) temperature-dependent and (2) accompanied by increases in respiration rate and, in some species, light sensitivity (Supplementary Table S3). Li et al. [35] reported a correlation between imbibition rate and germination rate, as in the present study, which also found a correlation between imbibition rate and germination-stage drought tolerance, as reported previously ([29,41], Supplementary Table S3).

Papilla shape, length, and density can be used as distinguishing taxonomic characteristics. However, the observation of these traits using light microscopy is nearly impossible. In most cases, individual cells possess four to five papillae, which are distributed in a regular pattern along the axis of the cell. Young papillae are wider than mature papillae but are also shorter and undergo morphological changes as they mature, eventually developing into three-m to five-branched structures. Rashid et al. [42] suggested that testa papillae contribute to moisture retention, especially in the soil. In the present study, the No.P, APA, and PAR were all significantly positively correlated with 5-h water uptake rate, and *D_b_*TP was significantly negatively correlated with germination-stage drought tolerance (Tables 4,5).

**Table 4.** Drought tolerance parameters of 35 rapeseed (*Brassica napus* L.) germplasm accessions from China

Accession	Germination rate (control)	Germination rate (drought)	Seedling height (control)	Seedling height (drought)	Seedling fresh weight (control)	Seedling fresh weight (drought)	Relative germination rate (%)	Relative Seedling Height (%)	Drought tolerance index
1	85.0	72.0	3.40	1.55	0.039	0.027	84.7	45.6	0.39
3	96.5	92.5	2.60	1.65	0.029	0.021	95.9	63.5	0.61
4	91.5	86.5	3.45	2.65	0.035	0.027	94.5	76.8	0.73
5	92.0	81.0	3.60	2.70	0.042	0.025	88.0	75.0	0.66
6	76.5	65.5	2.55	2.20	0.040	0.037	85.6	86.3	0.74
7	75.5	61.0	3.70	2.15	0.049	0.027	80.8	58.1	0.47
10	91.0	80.5	3.10	1.60	0.040	0.023	88.5	51.6	0.46
12	97.5	94.5	3.35	2.45	0.049	0.022	96.9	73.1	0.71
13	95.5	89.0	2.95	2.60	0.040	0.030	93.2	88.1	0.82
14	94.5	86.0	3.75	3.50	0.040	0.032	91.0	93.3	0.85
16	97.5	83.3	3.40	2.62	0.034	0.022	85.4	77.2	0.66
17	99.0	84.6	3.08	2.31	0.028	0.030	85.4	75.0	0.64
18	99.0	83.7	3.68	2.92	0.044	0.038	84.6	79.3	0.67
19	95.0	82.5	3.73	2.82	0.050	0.032	86.8	75.6	0.66
20	98.0	83.7	3.22	2.72	0.037	0.031	85.4	84.6	0.72
21	98.0	85.0	3.21	2.84	0.035	0.029	86.7	88.5	0.77
22	99.5	83.7	3.14	3.00	0.037	0.034	84.2	95.7	0.81
24	97.0	84.6	4.15	2.79	0.035	0.025	87.2	67.2	0.59
25	89.5	82.5	3.92	2.44	0.049	0.025	92.1	62.1	0.57
27	98.5	83.3	3.39	2.41	0.039	0.030	84.6	70.9	0.6
28	98.5	84.6	4.83	2.45	0.040	0.022	85.9	50.6	0.43
32	95.5	79.1	2.82	1.82	0.051	0.035	82.8	64.5	0.53
33	99.0	82.5	3.00	2.16	0.037	0.028	83.3	72.0	0.60
34	98.5	83.3	2.81	2.19	0.042	0.030	84.6	78.1	0.66
36	98.5	85.0	3.45	2.32	0.042	0.030	86.3	67.3	0.58
37	99.0	84.6	3.56	2.10	0.040	0.028	85.4	58.9	0.50
39	97.5	81.2	4.16	3.09	0.042	0.032	83.3	74.2	0.62
40	100.0	84.2	3.09	2.74	0.039	0.0033	84.2	88.7	0.75
42	97.0	83.7	3.68	2.34	0.041	0.027	86.3	63.7	0.55
43	98.5	83.3	3.54	2.37	0.035	0.021	84.6	67.0	0.57
44	94.0	79.9	4.05	3.15	0.034	0.026	85.0	77.7	0.66
45	99.5	85.0	3.16	2.59	0.035	0.027	84.5	85.7	0.72
47	98.0	83.7	3.03	2.27	0.051	0.032	85.4	75.0	0.64
48	96.5	82.0	2.60	1.4	0.035	0.016	85.0	70.0	0.59
49	97.0	83.7	3.18	2.70	0.051	0.038	86.3	84.8	0.73
	95.2	82.6	3.38	2.44	0.040	0.028	86.7	73.3	0.64
*B*_RGB_	−0.37*	−0.44**	−0.34*	−0.29	0.13	0.29	−0.23	−0.03	−0.09
*H*_HSB_	−0.47**	−0.36*	−0.25	−0.39*	0.07	0.05	0.06	−0.26	−0.23
*S*_HSB_	0.36*	0.43*	0.34*	0.3	−0.15	−0.29	0.22	0.04	0.1
*B*_HSB_	−0.46**	−0.31	−0.22	−0.39*	0.04	0.01	0.11	−0.3	−0.26
L*	−0.41*	−0.39*	−0.25	−0.4*	−0.04	0.05	−0.1	−0.24	−0.26
B*	0.3	0.39*	0.32	0.17	−0.24	−0.37*	0.24	−0.08	−0.01
*M*	0.38*	0.33	0.25	0.40*	0.01	−0.06	0.03	0.25	0.25
*Y*	0.35*	0.44**	0.38*	0.25	−0.11	−0.33*	0.24	−0.05	0.01
*D*_g_	−0.57**	−0.43**	−0.07	−0.3	0.01	−0.07	0.06	−0.29	−0.25
*D*_b_	−0.49**	−0.40*	−0.20	−0.33*	0.03	−0.02	0.02	−0.22	−0.21
*D*_g_P	−0.43*	−0.31	0.02	−0.09	0.03	−0.07	0.08	−0.13	−0.1
*D*_b_TP	−0.32	−0.31	0.01	−0.34*	−0.02	−0.02	−0.09	−0.40*	−0.39*

(*P < 0.05, ** *P < *0.01) EC:electric conductivity, WI:weight increase; *H*_HSB,_ hue of testa; *B*_HSB,_ brightness of testa; *S*_HSB_, S of testa, *B*_RGB,_ B value of RGB; *G*_RGB,_G value of RGB; L*, lightness; *a*, from magenta to green; b*, from yellow to blue; *M*, magenta; *Y*, yellow *IOD*, integrated optical density; *D*_r_P, red channel gray value of papilla; *D*_g_P green channel gray value of papilla; *D*_b_TP, blue channel gray value of total papilla; *D_g_*, green channel gray value of testa; *D*_b_, blue channel gray value of testa.

**Table 5.** Eigenvalues and contribution rate of principal components

Parameters	PC1	PC2	PC3
*H*_HSB_	0.326	0.166	−0.101
*B*_HSB_	0.319	0.155	−0.060
*S*_HSB_	−0.336	0.274	0.008
*B*_RGB_	0.332	−0.288	0.021
*L*	0.320	−0.061	0.215
*b*	−0.286	0.339	0.159
*M*	−0.331	0.121	−0.155
*Y*	−0.329	0.230	0.019
*D*_g_	0.201	0.415	−0.073
*D*_b_	0.033	0.112	0.927
*D*_g_P	0.250	0.475	0.005
*D*_b_TP	0.251	0.441	−0.161
Eigen values	7.426	1.887	1.058
Contribution rate (%)	61.884	15.723	8.820
Cumulative contribution rate (%)	61.884	77.608	86.428

*H*_HSB,_ Hue of testa; *B*_HSB,_ Brightness of testa; *S*_HSB_, S of testa, *B*_RGB,_ B value of RGB; *L*,lightness; *a*, from magenta to green; *b*, from yellow to blue; *M*, magenta;*Y*,yellow;*D*_g_P green channel gray value of papilla; *D*_b_TP, blue channel gray value of total papilla; *D_g_*, green channel gray value of; *D_b_*, blue channel gray value of testa.

Previous studies of other crop species have demonstrated that testa color affects seed germination rate, emergence part, seedling dry weight, and water uptake percentage. For example, black guar (*Cyamopsis tetragonoloba* L. Taub) seeds exhibit higher water uptake and germination rates than light-colored guar seeds [43]. In *Atriplex cordobensis*, reddish and light-brown seeds exhibit higher germination ratios and seedling dry weights than dark-brown seeds [44], and in red clover, yellow seed lots exhibit the highest germination and emergence ratios and most rapid germination [44].

### Principal component analysis and electric conductivity

Principal component analysis of the 12 microscopic color and morphology traits indicated that the 35 rapeseed accessions could be classified as drought tolerant or drought resistant. However, the PCA results of some accessions (e.g., 1, 07, 13, and 21) were inconsistent with the DTI [41], possibly owing to the selection of evaluation indices or to the selection of indices for PCA. Plant breeders around the world have been trying to develop yellow-seeded *B. napus* genotypes using crosses that involve naturally occurring yellow-seeded *Brassica* species [45]. Consequently, most research efforts have focused on the characteristics of different genotypes with testa colors in *B. napus* and molecular markers associated with seed color traits

As previously reported by Mandizvo and Odindo [13], the present study found that black, red, or LSP seeds exhibited lower rates of exudate leakage, whereas yellow and LBP seeds are more conducive to water entry, resulting in more rapid water update and, possibly, imbibition damage [13]. Previous studies have associated reductions in germination ability with membrane deterioration, as measured by electrical conductivity and electrolyte leakage [16]. Landraces differed significantly (*P* < 0.001) in testa color, testa thickness, electrical conductivity, hydration rate, germination rate, and seedling length. The electrical conductivity of the seed leachate increased with increased duration.

## Conclusion

The aim of the present study was to investigate the relationship between microscopic testa color and papilla characteristics, imbibition, and germination-stage drought tolerance in rapeseed. Analysis indicated that both microscopic color and papilla traits were correlated with seed imbibition, seed germination, seedling growth, and germination-stage drought tolerance. However, additional research is needed to determine the composition of testa color and mechanism(s) by which papilla structure affects seed imbibition, seed germination, seedling growth, and germination-stage drought tolerance.

## Supplementary Material

Supplemental Material
