# Supplementary material for: Effects of microscopic testa color and morphologyon the water uptake ability and drought tolerance of germination-stage rapeseed (Brassica napus L.)
Source: Bioengineered. 2021 Dec 24;12(2):9341–55. doi: 10.1080/21655979.2021.2000789 (PMC8809997; doi:10.1080/21655979.2021.2000789)
Supplement: Supplemental Material [file KBIE_A_2000789_SM6176.docx]

**Table S1. Different responses of drought tolerance characteristics of 35 accessions after 24h PEG treatment.**

| Accession | Germination  rate on distilled water(%) | Germination  rate on 10%PEG(%) | Seedling  height of CK | Seedling  height of 10%PEG | Seedling fresh weight of CK | Seedling fresh weight of 10%PEG | Relative germination  rate(%) | Relative  Seedling  height (%) | DTI of  seedling  height |
| --- | --- | --- | --- | --- | --- | --- | --- | --- | --- |
| 9H01 | 85.0 | 72.0 | 3.40 | 1.55 | 0.039 | 0.027 | 84.7 | 45.6 | 0.39 |
| 9H03 | 96.5 | 92.5 | 2.60 | 1.65 | 0.029 | 0.021 | 95.9 | 63.5 | 0.61 |
| 9H04 | 91.5 | 86.5 | 3.45 | 2.65 | 0.035 | 0.027 | 94.5 | 76.8 | 0.73 |
| 9H05 | 92.0 | 81.0 | 3.60 | 2.70 | 0.042 | 0.025 | 88.0 | 75.0 | 0.66 |
| 9H06 | 76.5 | 65.5 | 2.55 | 2.20 | 0.040 | 0.037 | 85.6 | 86.3 | 0.74 |
| 9H07 | 75.5 | 61.0 | 3.70 | 2.15 | 0.049 | 0.027 | 80.8 | 58.1 | 0.47 |
| 9H10 | 91.0 | 80.5 | 3.10 | 1.60 | 0.040 | 0.023 | 88.5 | 51.6 | 0.46 |
| 9H12 | 97.5 | 94.5 | 3.35 | 2.45 | 0.049 | 0.022 | 96.9 | 73.1 | 0.71 |
| 9H13 | 95.5 | 89.0 | 2.95 | 2.60 | 0.040 | 0.030 | 93.2 | 88.1 | 0.82 |
| 9H14 | 94.5 | 86.0 | 3.75 | 3.50 | 0.040 | 0.032 | 91.0 | 93.3 | 0.85 |
| 9H16 | 97.5 | 83.3 | 3.40 | 2.62 | 0.034 | 0.022 | 85.4 | 77.2 | 0.66 |
| 9H17 | 99.0 | 84.6 | 3.08 | 2.31 | 0.028 | 0.030 | 85.4 | 75.0 | 0.64 |
| 9H18 | 99.0 | 83.7 | 3.68 | 2.92 | 0.044 | 0.038 | 84.6 | 79.3 | 0.67 |
| 9H19 | 95.0 | 82.5 | 3.73 | 2.82 | 0.050 | 0.032 | 86.8 | 75.6 | 0.66 |
| 9H20 | 98.0 | 83.7 | 3.22 | 2.72 | 0.037 | 0.031 | 85.4 | 84.6 | 0.72 |
| 9H21 | 98.0 | 85.0 | 3.21 | 2.84 | 0.035 | 0.029 | 86.7 | 88.5 | 0.77 |
| 9H22 | 99.5 | 83.7 | 3.14 | 3.00 | 0.037 | 0.034 | 84.2 | 95.7 | 0.81 |
| 9H24 | 97.0 | 84.6 | 4.15 | 2.79 | 0.035 | 0.025 | 87.2 | 67.2 | 0.59 |
| 9H25 | 89.5 | 82.5 | 3.92 | 2.44 | 0.049 | 0.025 | 92.1 | 62.1 | 0.57 |
| 9H27 | 98.5 | 83.3 | 3.39 | 2.41 | 0.039 | 0.030 | 84.6 | 70.9 | 0.6 |
| 9H28 | 98.5 | 84.6 | 4.83 | 2.45 | 0.040 | 0.022 | 85.9 | 50.6 | 0.43 |
| 9H32 | 95.5 | 79.1 | 2.82 | 1.82 | 0.051 | 0.035 | 82.8 | 64.5 | 0.53 |
| 9H33 | 99.0 | 82.5 | 3.00 | 2.16 | 0.037 | 0.028 | 83.3 | 72.0 | 0.60 |
| 9H34 | 98.5 | 83.3 | 2.81 | 2.19 | 0.042 | 0.030 | 84.6 | 78.1 | 0.66 |
| 9H36 | 98.5 | 85.0 | 3.45 | 2.32 | 0.042 | 0.030 | 86.3 | 67.3 | 0.58 |
| 9H37 | 99.0 | 84.6 | 3.56 | 2.10 | 0.040 | 0.028 | 85.4 | 58.9 | 0.50 |
| 9H39 | 97.5 | 81.2 | 4.16 | 3.09 | 0.042 | 0.032 | 83.3 | 74.2 | 0.62 |
| 9H40 | 100.0 | 84.2 | 3.09 | 2.74 | 0.039 | 0.0033 | 84.2 | 88.7 | 0.75 |
| 9H42 | 97.0 | 83.7 | 3.68 | 2.34 | 0.041 | 0.027 | 86.3 | 63.7 | 0.55 |
| 9H43 | 98.5 | 83.3 | 3.54 | 2.37 | 0.035 | 0.021 | 84.6 | 67.0 | 0.57 |
| 9H44 | 94.0 | 79.9 | 4.05 | 3.15 | 0.034 | 0.026 | 85.0 | 77.7 | 0.66 |
| 9H45 | 99.5 | 85.0 | 3.16 | 2.59 | 0.035 | 0.027 | 84.5 | 85.7 | 0.72 |
| 9H47 | 98.0 | 83.7 | 3.03 | 2.27 | 0.051 | 0.032 | 85.4 | 75.0 | 0.64 |
| 9H48 | 96.5 | 82.0 | 2.60 | 1.4 | 0.035 | 0.016 | 85.0 | 70.0 | 0.59 |
| 9H49 | 97.0 | 83.7 | 3.18 | 2.70 | 0.051 | 0.038 | 86.3 | 84.8 | 0.73 |
| mean | 95.2 | 82.6 | 3.38 | 2.44 | 0.040 | 0.028 | 86.7 | 73.3 | 0.64 |
| *F* value |  |  |  |  |  |  |  |  |  |
| *B*_RGB_ | -0.37^*^ | -0.44^**^ | -0.34^*^ | -0.29 | 0.13 | 0.29 | -0.23 | -0.03 | -0.09 |
| *H*_HSB_ | -0.47^**^ | -0.36^*^ | -0.25 | -0.39^*^ | 0.07 | 0.05 | 0.06 | -0.26 | -0.23 |
| *S*_HSB_ | 0.36^*^ | 0.43^*^ | 0.34^*^ | 0.3 | -0.15 | -0.29 | 0.22 | 0.04 | 0.1 |
| *B*_HSB_ | -0.46^**^ | -0.31 | -0.22 | -0.39^*^ | 0.04 | 0.01 | 0.11 | -0.3 | -0.26 |
| *L* | -0.41^*^ | -0.39^*^ | -0.25 | -0.4^*^ | -0.04 | 0.05 | -0.1 | -0.24 | -0.26 |
| *b* | 0.3 | 0.39^*^ | 0.32 | 0.17 | -0.24 | -0.37^*^ | 0.24 | -0.08 | -0.01 |
| *M* | 0.38^*^ | 0.33 | 0.25 | 0.40^*^ | 0.01 | -0.06 | 0.03 | 0.25 | 0.25 |
| *Y* | 0.35^*^ | 0.44^**^ | 0.38^*^ | 0.25 | -0.11 | -0.33^*^ | 0.24 | -0.05 | 0.01 |
| *D*_g_ | -0.57^**^ | -0.43^**^ | -0.07 | -0.3 | 0.01 | -0.07 | 0.06 | -0.29 | -0.25 |
| *D*_b_ | -0.49^**^ | -0.40^*^ | -0.20 | -0.33^*^ | 0.03 | -0.02 | 0.02 | -0.22 | -0.21 |
| *D*_g_P | -0.43^*^ | -0.31 | 0.02 | -0.09 | 0.03 | -0.07 | 0.08 | -0.13 | -0.1 |
| *D*_b_TP | -0.32 | -0.31 | 0.01 | -0.34^*^ | -0.02 | -0.02 | -0.09 | -0.40^*^ | -0.39^*^ |

Note:(^*^*P<*0.05, ^**^ *P<*0.01) EC:electric conductivity, WI:weight increase;*H*_HSB,_ Hue of testa; *B*_HSB,_ Brightness of testa; *S*_HSB_, S of testa, *B*_RGB,_ B value of RGB; *G*_RGB,_G value of RGB;*L*, lightness; *a*, from magenta to green; *b*, from yellow to blue; *M*, magenta;*Y*,yellow *IOD*, integrated optical density; *D*_r_P, red channel gray value of papilla; *D*_g_P green channel gray value of papilla; *D*_b_TP, blue channel gray value of total papilla; *D_g_*, green channel gray value of testa; *D_b_,* blue channel gray value of testa.

**Table S2: Eigenvalues and contribution rate of principal components**

| Parameters | PC1 | PC2 | PC3 |
| --- | --- | --- | --- |
| *H*_HSB_ | 0.889 | 0.228 | -0.104 |
| *B*_HSB_ | 0.870 | 0.213 | -0.062 |
| *S*_HSB_ | 0.904 | -0.396 | 0.022 |
| *B*_RGB_ | -0.915 | 0.377 | 0.008 |
| *L^*^* | 0.871 | -0.084 | 0.221 |
| *b^*^* | -0.780 | 0.466 | 0.163 |
| *M* | -0.902 | 0.167 | -0.159 |
| *Y* | -0.895 | 0.316 | 0.011 |
| *D*_g_ | 0.549 | 0.570 | -0.075 |
| *D*_b_ | 0.089 | 0.154 | 0.954 |
| *D*_g_P | 0.682 | 0.652 | 0.005 |
| *D*_b_TP | 0.684 | 0.605 | -0.165 |
| Eigen values | 7.426 | 1.887 | 1.058 |
| Contribution rate (%) | 61.884 | 15.723 | 8.820 |
| Cumulative contribution rate (%) | 61.884 | 77.608 | 86.428 |

Note: *H*_HSB,_ Hue of testa; *B*_HSB,_ Brightness of testa; *S*_HSB_, S of testa, *B*_RGB,_ B value of RGB; *L*, lightness; *a*, from magenta to green; *b*, from yellow to blue; *M*, magenta; *Y*, yellow; *D*_g_P green channel gray value of papilla; *D*_b_TP, blue channel gray value of total papilla; *D_g_*, green channel gray value of; *D_b_*, blue channel gray value of testa.

**Table S3. Highest drought tolerance index in different studies**

| **Drought tolerance index** | **Condition** | **Result** | **Reference** |
| --- | --- | --- | --- |
| drought tolerance index of seedling height | PEG stress | 35 genotypes were identified | this study |
| germination rate, germ dry weight and storage material transport rate | Soil Stress | 4 of the thirty-four hybrids were identified as drought tolerant | 47 |
| physiological and biochemical parameters viz., seed morphology, seed germination, length, fresh weight, dry weight and proline accumulation | PEG stress | 51 rice genotypes were identified which can be used as a source of genomic approach | 44 |
| germination rate, fast seedling growth and early vigor under drought | PEG stress | BS17 showed high germination rate, fast seedling growth and early vigor under drought. | 41 |
| the germination stage including seed germination stress index, relative germination rate, radicle / plumule ratio, root volume | PEG stress | 10 tested varieties were divided into 3 groups with high, moderate and low drought resistance respectively. | 46 |
| total germination，speed of germination，speed of accumulated germination， coefficient of germination rate | PEG stress | 51 diverse open-pollinated maize populations increase levels of drought at germination, Acala 1517-99 as the most drought-tolerant line | 41 |
| proline, soluble protein and malondialdehyde contents | PEG stress | 74 lines of new-type *Brassica napus* were identified | 43 |
| promptness, germination, seedling height, fresh weight, dry weight and stress indexes | PEG stress | sixty sunflower accessions to drought stress at germination， The accessions 017583, A-75, A-79, 017592, G-33, A-48, A-23, G-61, HBRS-1 and 017566 were selected as drought tolerant | 42 |
| the germination rate, germination index, germination potential germination osmotic resistance index，SOD | PEG stress | PI401470 (No.12), PI401477 (No.16), PI470942 (No.23) and PI544021 (No.28) were identified as highly osmotic resistant cultivars. | [45](http://www-webofscience-com-s.webvpn.ahau.edu.cn:8800/wos/alldb/full-record/WOS:000460430000026) |
